# Development of ankylosing spondylitis in patients with ulcerative colitis: A systematic meta-analysis

**DOI:** 10.1371/journal.pone.0289021

**Published:** 2023-08-01

**Authors:** Aitao Lin, Yongyi Tan, Jinxia Chen, Xiaoyu Liu, Jinyu Wu

**Affiliations:** 1 Guangxi University of Traditional Chinese Medicine, Nanning, Guangxi, China; 2 Department of Rheumatology, The First Affiliated Hospital of Guangxi University of Chinese Medicine, Nanning, Guangxi, China; Cairo University Kasr Alainy Faculty of Medicine, EGYPT

## Abstract

**Background:**

Ulcerative colitis (UC) is a manifestation of inflammatory bowel disease (IBD), which can cause inflammation of the intestinal tract. Ankylosing spondylitis (AS) is an inflammatory disease of the sacroiliac joints. Many studies have found that some UC patients progress to AS. In this study, we conducted a literature search and meta-analysis to investigate the prevalence of AS among UC patients during follow-up.

**Methods:**

The studies related to the AS among patients with UC were obtained from PubMed, Web of Science, Embase, and Cochrane Library databases since its inception-December 2022. The literature was screened strictly according to inclusion and exclusion criteria. Forest plots were used to detect the overall incidence of AS in UC and to compare the risk ratios for the development of AS in the UC. The heterogeneity of studies was assessed using I^2^ statistical methods.

**Results:**

1) 17 studies with 98704 UC patients were included. 2)700 UC patients developed AS during follow-up (1.66%, 95% CI: 0.89–2.62%). Human leukocyte antigen B27 (HLA-B27) was reported in 3 studies. HLA-B27 positivity was significantly higher than the incidence of HLA-B27 negativity in AS patients (68.29% vs 31.71%, P < 0.0001). There was significantly increased risk of AS development in HLA-B27 positive IBD patients (RR: 22.17, 95% CI: 11.79–41.66, P < 0.0001). 3)The definite follow-up time was reported in 12 studies (range: 0.3–40 years). After follow-up for ≥5 years, the incidence of AS among patients with UC was 1.75% (95% CI: 0.62–3.37%). Meanwhile, after follow-up for <5 years, the incidence of AS among patients with UC was 1.41% (95% CI: 0.65–2.37%) which was significant.

**Conclusion:**

Patients with UC are more likely to develop AS in the future. Furthermore, the IBD patients are at a higher risk of AS who have positive HLA-B27. The incidence of AS increased with longer follow-up time.

## 1. Introduction

Ulcerative colitis (UC) is a form of inflammatory bowel disease (IBD)—an inflammatory disease of the gastrointestinal tract. In short, UC is a chronic autoimmune-related inflammatory disease. The clinical manifestations of UC mainly involve abdominal pain, diarrhea, and hematochezia [1]. It has long been recognized that UC may affect multiple organs of the body. The involvement of organs outside the gastrointestinal tract is often referred to as extraintestinal manifestations (EIMs) of UC, which mainly involves the hepatobiliary tract and the musculoskeletal system [2]. The literature suggests that the etiology of EIMs involves interactions between the environment and immune system [3]. Moreover, EIMs may be associated with the dysfunction of the intestinal flora and intestinal immune response [4]. Past studies have reported that approximately 30% of UC patients present with extra-intestinal manifestations such as the skeletal muscles and skin. Nevertheless, the pathogenesis of EIMs is unclear.

Ankylosing spondylitis (AS) is a chronic autoimmune disease that usually affects the sacroiliac joints and spinal attachments. Clinically, AS is characterized by inflammatory pain and stiffness of the back [5]. AS may involve axial or peripheral joints. According to past studies, 6% of AS is reported in adult UC patients, although it is rare among children [6–8]. Consistently, several AS patients have subclinical IBDs [9]. Similarly, the occurrence of AS among patients with UC is reportedly approximately 3% [10]. Considering that the UC and AS may manifest with similar pathogenesis, it is critical to explore the causal relationship between them to improve the level of diagnosis and treatment.

Human leukocyte antigen B27 (HLA-B27) is a characteristic test indicator for AS. However, the specificity and sensitivity of AS patients with HLA-B27 has been reportedly to be low in the presence of IBD [11]. Furthermore, the duration from the onset to diagnosis of AS can take several years. Therefore, early diagnosis of AS among patients with UC is of great significance. In this study, we conducted an extensive literature search and meta-analysis to investigate the prevalence of AS among UC patients during their follow-up period so as to provide an evidence-based basis for clinical practice.

## 2. Material and methods

The Preferred Reporting Items for Systematic Reviews and Meta-Analyses (PRISMA) guidelines was used to guidance the systematic meta-analysis [12].

### 2.1 Sources of information

The literatures about the development of AS in UC patients were collected from four foreign databases, which included PubMed, The Cochrane Library, Web of Science, and Embase since its inception-December 2022. In addition to, we also collected the relevant articles from conference abstracts and references.

### 2.2 Search strategy

**Fig 1:**
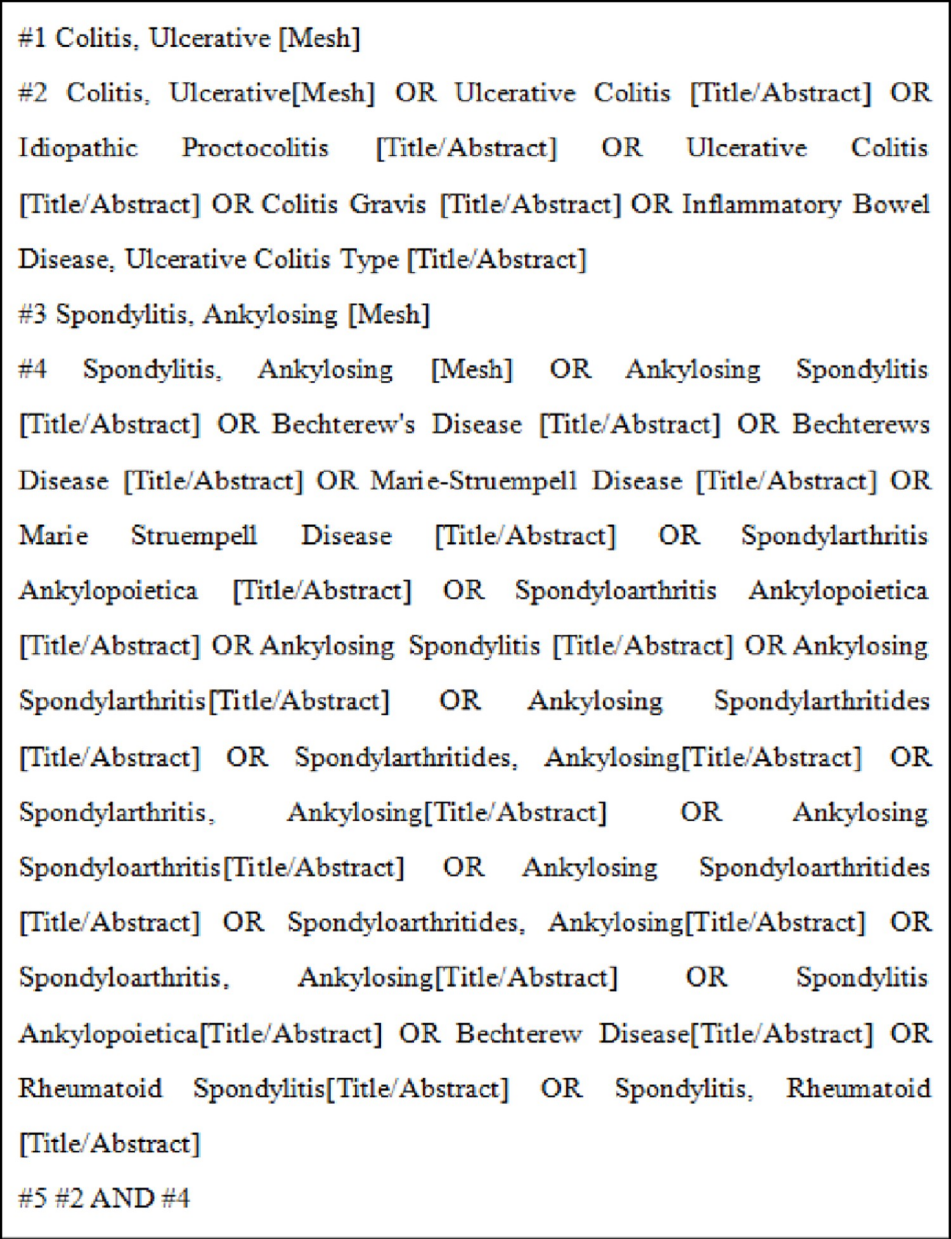
Search strategy of PubMed.

“Ulcerative Colitis”, “Idiopathic Proctocolitis”, “Ankylosing Spondylitis”, “Bechterew’s Disease” and so on were used as subject terms to search the relevant literature. We used the following search strategy for the PubMed database (Fig 1. Search strategy of PubMed.).

### 2.3 Inclusion criteria

#### 2.3.1 Object of study

Studies were included that patients with a clear diagnosis of UC who developed AS during follow-up. Used the 2010 Asia-Pacific Consensus [13] to diagnose UC patients. The 1984 modified New York criteria [14] was used to diagnose AS patients.

#### 2.3.2 Types of research and language of publication

English language literature (including cross-sectional studies, cohort studies, case-control studies, etc.) on the occurrence of AS during UC follow-up were included.

### 2.4 Exclusion criteria

① No follow-up study.
② Reviews, case reports, animal and cell-based experiments, etc.).
③ Repeatedly included literature, literature with incomplete data indicators or where complete information was not available.

### 2.5 Data extraction

#### 2.5.1 Literature screening and data extraction

Two reviewers (TYY, CJX) independently screened the literature strictly according to the inclusion and exclusion criteria and extracted data by a standardized form. The literature was managed using NoteExpress software. Initially, after eliminating duplicates for inclusion, the primary screening was done by reading the title and abstract of the literature. And then full-text literature was analyzed in detail. If there was disagreement between the 2 reviewers in the literature screening or data extraction process, the decision was submitted to a 3rd reviewers (WJY).

#### 2.5.2 Assessment of the quality of the literature

Case-control studies and cohort studies were evaluated for methodological quality using the Newcastle-Ottawa Scale (NOS) [15]. Cross-sectional studies using the AHRQ criteria. If 2 reviewers disagreed on the assessment of the quality of the literature, the decision was submitted to a 3rd reviewers (WJY). All included literature was of moderate to high quality.

### 2.6 Statistical analysis

The frequency of AS development in UC during follow-up was detected by a fixed or random-effects model. Literature heterogeneity was analysed using Revman 5.3 software and the R Statistical Software meta package. If *P* ≥ 0.10 and I^2^ ≤ 50%, the data were homogeneous across studies and were analysed using a fixed effects model. If *P*< 0.10 and/or I^2^ > 50%, we need to find the source of heterogeneity, or in cases where the source of heterogeneity was unclear, a random-effects model was used [16]. Besides, we estimated the risk ratio (RR) and its 95% confidence interval (CI) after followup for the AS development in UC patients. Using forest plots to summarize the data. Revman 5.3 software was used to draw funnel plots to evaluate publication bias.

## 3. Results

### 3.1 PROSPERO registration

Registration number: CRD42023405366.

### 3.2 Ethical approval

Our study did not involve human or animal experiments, which simply integrated results from other articles. Therefore, the study did not need to obtain ethical approval.

### 3.3 Literature search results

**Fig 2:**
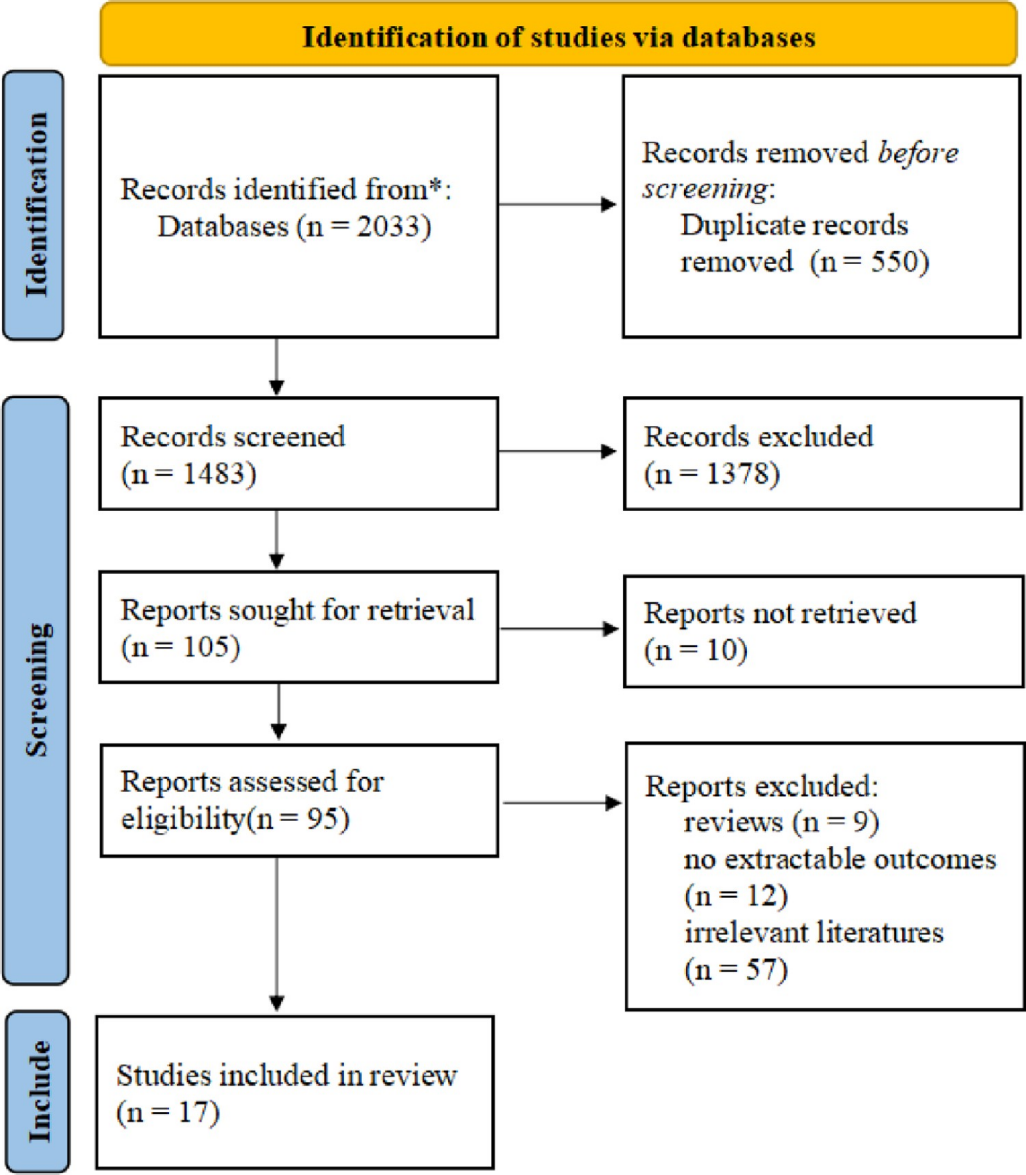
Literature screening process and results. Note: *Databases searched and literature obtained are as follows: PubMed (n = 554), Web of Science (n = 497), The Cochrane Library (n = 68), Embase (n = 914).

The databases search yielded 2033 relevant studies, and 17 studies were finally included for meta-analysis in strict accordance with the inclusion and exclusion criteria of this study (Fig 2. Literature screening process and results.) [17–33].

### 3.4 Basic characteristics of the included studies

17 studies were included, which 2 studies were paediatric studies and the rest were adult studies. A total of 98704 UC patients were followed up (Table 1. Studies included in the meta-analysis.).

**Table 1:** Studies included in the meta-analysis.

First author/Date	Country/Population	Years	UC/n	UC-AS/n	HLA-B27+/n (AS)	Followup period
**ØYVIND PALM/2001**	Norway/A	1990–1993	273	7	11	5yrs.
**Carol B/1974**	NA/P	1960–1970	86	3	NA	10yrs.(md)
**Raina Shivashankar/2014**	USA/A	1970–2004	362	1	NA	40yrs.
**Ignacio Marin-Jimenez/2015**	Spain/A	2008–2010	130	2	NA	25mo
**Jesús K/2017**	Mexican/A	2014–2015	154	8	NA	NA
**Alvilde M. Ossum/2017**	Norway/A	1990–1993	314	13	12	20yrs.
**Morten L Halling/2017**	Danish/A	1977–2013	31066	201	NA	NA
**Hoda M Malaty/2017**	USA/A	1996–2009	357	7	NA	5.6yrs.(md)
**Johan Burisch/2019**	Denmark/A	2007–2016	8000	16	NA	5yrs.
**Aimee Hiller/2019**	Swiss/A	2016	1438	44	NA	13yrs.(md)
**Hyo-Jeong Jang/2021**	S Korean/P	2010–2017	35	0	NA	3.2yrs.(md)
**C. Corrado/2011**	Italian/A	2000–2010	593	8	NA	40.7 mo (md)
**K. N. JALAN/1970**	England/A	1950–1967	399	17	NA	NA
**C. Salvarani/2001**	Italy、The Netherlands/A	1991–1993	98	2	5	50.3 mo(md)
**Jung Min Bae/2016**	S Korean/A	2009–2013	28197	130	NA	NA
**Asli Beslek/2009**	Turkish/A	2005.04–2005.08	94	6	NA	4mo(md)
**Timothy R. Card/2016**	England/A	1987–2011	27108	235	NA	NA

### 3.5 Meta-analysis

#### 3.5.1 Meta-analysis of the prevalence of AS in UC patients during follow-up

**Fig 3:**
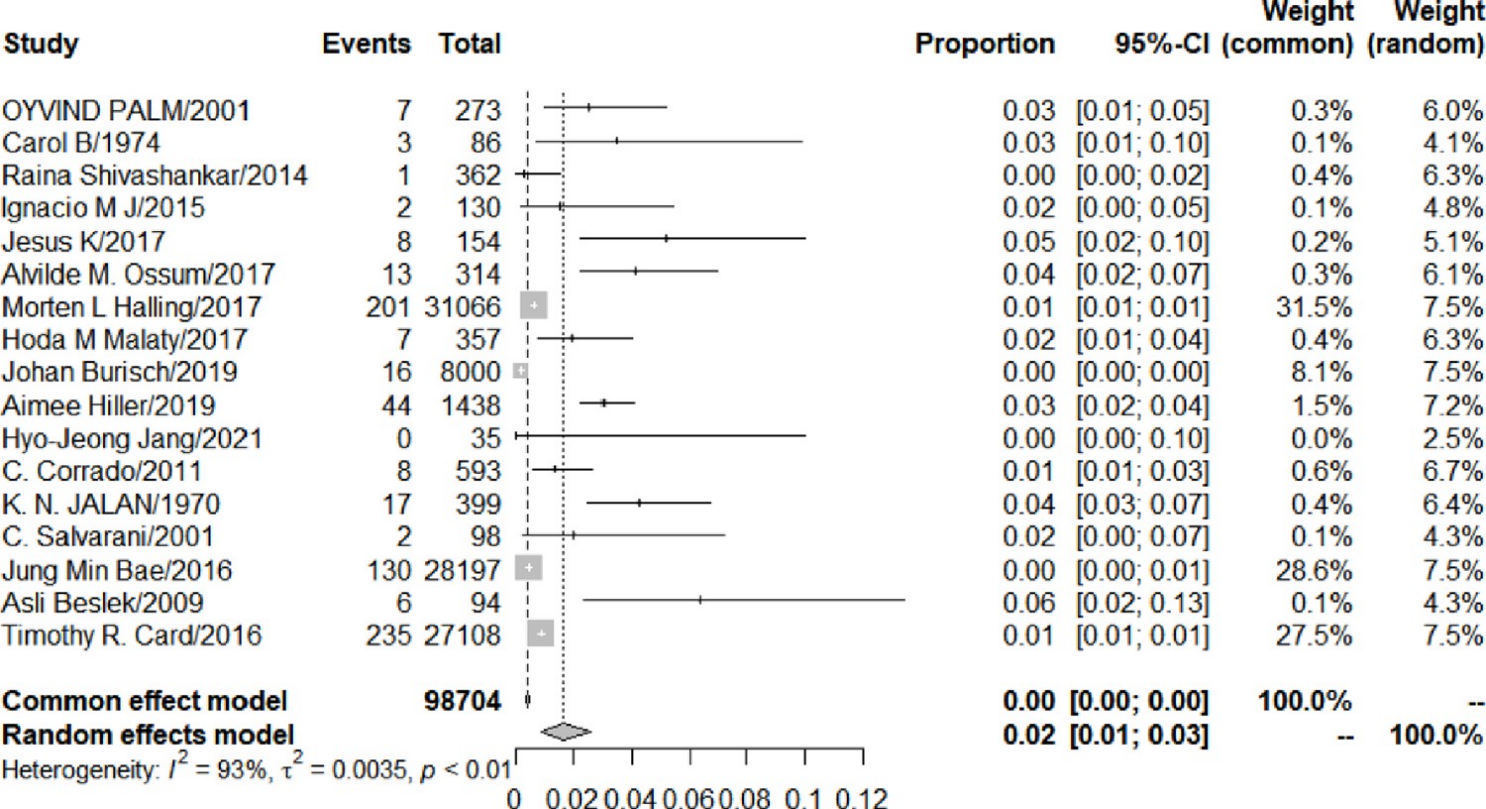
Forest plot of the meta-analysis of prevalence of AS among UC patients.

The prevalence of AS among UC patients was detected by a random effects model. During the follow-up, 700 UC patients developed AS. The meta-analysis result showed that the prevalence of AS among UC patients was 1.66% (95% CI: 0.89–2.62%). But the heterogeneity was at a high level (I2: 93%). Due to the relatively small number of articles, data could not be removed to eliminate heterogeneity, and the source of heterogeneity was unclear (Fig 3. Forest plot of the meta-analysis of prevalence of AS among UC patients.).

#### 3.5.2 Follow-up duration of UC and AS risk

**Fig 4:**
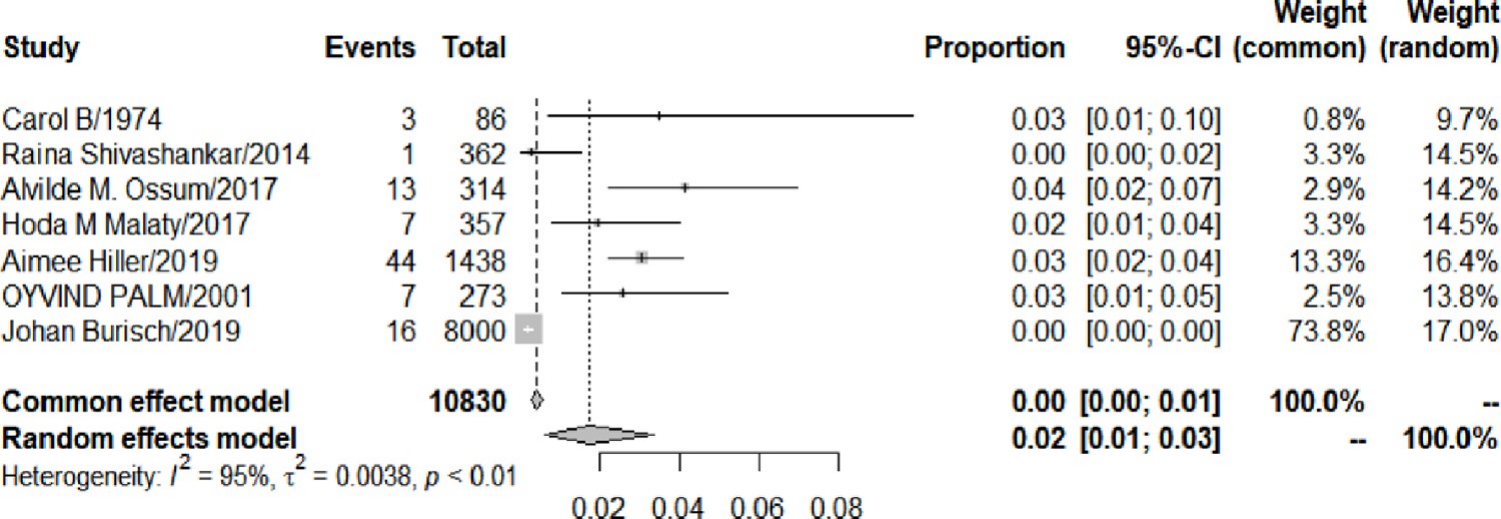
Forest plot of the meta-analysis of prevalence of AS among patients with UC with follow-up for ≥ 5years.

**Fig 5:**
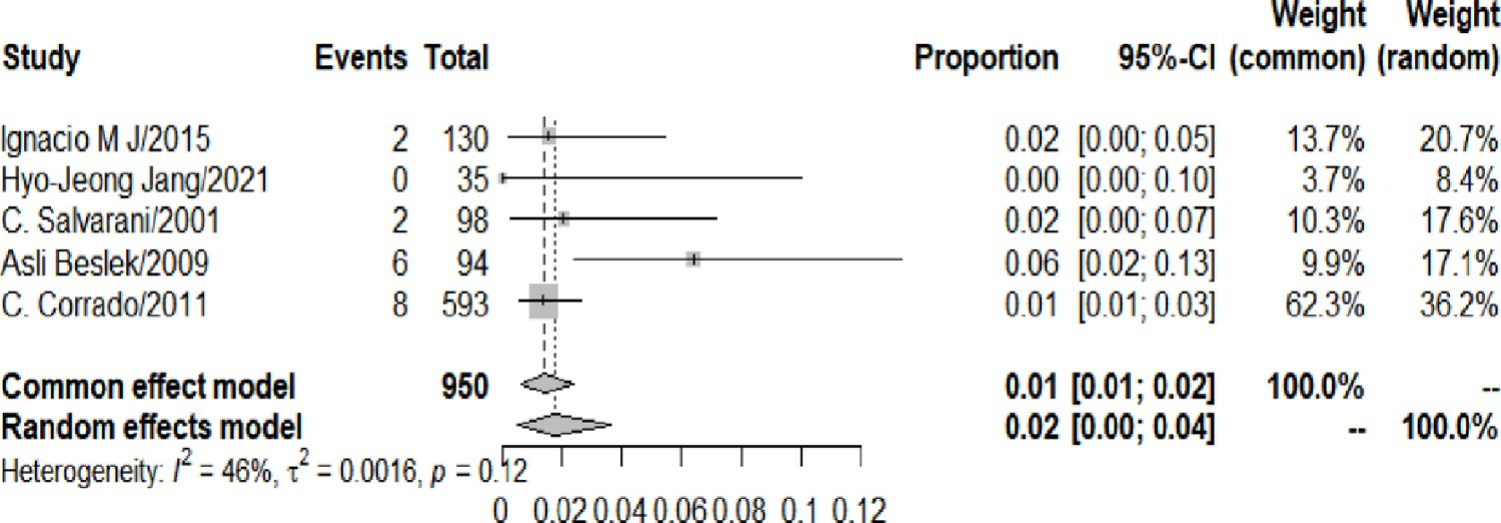
Forest plot of the meta-analysis of prevalence of AS among patients with UC with follow-up for ≤ 5years.

12 studies mentioned the follow-up duration of UC (range: 0.3–40 years). The follow-up time duration was ≥ 5 years in 7 studies, while the follow-up time duration was < 5 years in 5 studies. UC patients whose follow-up period ≥ 5 years were more likely to develop AS than those with follow-up period < 5 years (1.75% vs 1.41%). The risk of AS development among UC patients was significantly higher in studies that had a longer follow–up period (P<0.000) (Fig 4. Forest plot of the meta-analysis of prevalence of AS among patients with UC with follow-up for ≥ 5years.; Fig 5. Forest plot of the meta-analysis of prevalence of AS among patients with UC with follow-up for ≤ 5years.).

#### 3.5.3 HLA-B27 positivity and AS risk

**Fig 6:**
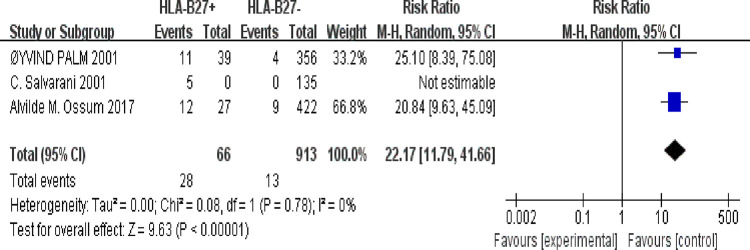
Forest plot of the risk ratios of AS development in IBD patients with HLA-B27. Note: the left of “Events” means “HLA-B27+AS+”, the left of “Total” means “HLA-B27+AS-”; the right of “Events” means “HLA-B27-AS+”, the right of “Total” means “HLA-B27-AS-”.

3 studies mentioned the HLA-B27 positivity which included 1033 IBD patients. The HLA-B27 positivity of 94 IBD patients were detected in study. HLA-B27 positivity was significantly higher than HLA-B27 negativity in AS patients (68.29% vs 31.71%, P < 0.0001). There was significantly increased risk of AS development in HLA-B27 positive IBD patients (RR. 22.17, 95% CI: 11.79–41.66, P < 0.0001) (Fig 6. Forest plot of the risk ratios of AS development in IBD patients with HLA-B27.).

## 4. Discussion

Our study results indicated that the prevalence of AS development in UC patients was 1.66%. Moreover, HLA-B27 positivity was significantly higher than HLA-B27 negativity in patients with AS (68.29% vs 31.71%, P < 0.0001), implying that HLA-B27 positivity is an important risk factor for the development of AS. On the other hand, the risk of AS development in UC patients with ≥5 years of follow-up was reported to be 1.75% (95% CI: 0.62–3.37%) and 1.41% (95% CI: 0.65–2.37%) in UC patients with <5 years of follow-up (P < 0.05), indicating that the risk of AS in UC patients may increase over time.

To the best of our knowledge, several studies have demonstrated a link between intestinal and joint inflammation [33–35]. Moreover, researchers have proposed the gut–joint axis hypothesis based on the pathogenesis of IBD and AS [36, 37]. Previously, Tito et al. reported a significant difference in the intestinal microbial composition between spondylarthritis patients without and with intestinal inflammation [38]. The gut microbes are an important aspect of the interaction between exogenous microbes and HLA-B27 [39]. Costello et al. concluded that dysbiosis of the gut flora may be a common pathogenesis of AS and intestinal inflammation [40]. In fact, 5%–10% of AS patients have concomitant IBD, and >70% of AS patients have subclinical intestinal inflammation. Consistently, a systematic review and meta-analysis revealed that AS occurs in 3% of patients with IBD [11]. In addition, up to 30% of patients with primary IBD develop joint symptoms, suggesting that the intestinal and joint inflammation may share a common pathogenesis.

HLA-B27 serves as the strongest genetic risk for AS, with 80–90% positivity reported in AS patients [40]. Ossum A. M. et al. concluded that the lower HLA-B27 positivity may affect the lower prevalence of AS [28]. Similarly, our study revealed that HLA-B27 positivity was significantly higher than HLA-B27 negativity in AS patients (68.29% vs 31.71%, P < 0.0001). Moreover, there was a significantly increased risk of AS development in HLA-B27-positive IBD patients (RR: 22.17, 95% CI: 11.79–41.66, P < 0.0001). However, HLA-B27 is less specific and less sensitive in patients with spondylitis presenting with IBD [11]. In clinical settings, more attention should be paid to HLA-B27 of IBD patients.

The follow-up duration is associated with the occurrence of AS in UC patients. The low prevalence of AS in the UC patients who were followed up may be attributed to the relatively short duration of follow-up. Our study results showed that, after follow-up for ≥5 years, the incidence of AS among patients with UC was 1.75% (95% CI: 0.62–3.37%). Meanwhile, at a follow-up for <5 years, the incidence of AS among patients with UC was 1.41% (95% CI: 0.65–2.37%) which was deemed significant. Moreover, a retrospective analyses conducted in Korea did not find the onset of AS during 3.2 years of follow-up among pediatric patients with UC [41]. In the present systematic meta-analysis, the incidence of AS was found to increase with the follow-up time. Similarly, Pamuk O N, et al concluded that the incidence of systemic lupus erythematosus increased with the follow-up time [42]. It can thus be concluded that the odds of developing AS in UC patients increases over time, which deserves the attention of clinicians.

There are, of course, limitations to our study. First, almost all included studies were retrospective case reports of AS in patients with UC. Second, the search literature was restricted to studies published in the English language. Many studies from other languages were not included in this study. Third, the therapy adopted for UC patients and their effects on AS development was also not studied. Finally, data collected on the gender or age of UC patients who developed AS was inadequate, which may have affected the calculation of the risk of developing AS in UC patients [43].

## 5. Conclusions

In patients with UC, the risk of developing AS is higher than in the general population. In addition, the HLA-B27 positive in IBD patients is a risk factor for AS which need follow-up. Finally, odds of developing AS in UC patients may increase over time.
